# The impact of cage dividers on mouse aggression, dominance and hormone levels

**DOI:** 10.1371/journal.pone.0297358

**Published:** 2024-02-07

**Authors:** Christina Streiff, Adrian Herrera, Bernhard Voelkl, Rupert Palme, Hanno Würbel, Janja Novak

**Affiliations:** 1 Animal Welfare Division, Vetsuisse Faculty, University of Bern, Bern, Switzerland; 2 Unit of Physiology, Pathophysiology, and Experimental Endocrinology, Department of Biomedical Sciences, University of Veterinary Medicine, Vienna, Austria; Kyoto University Graduate School of Informatics: Kyoto Daigaku Daigakuin Johogaku Kenkyuka, JAPAN

## Abstract

Home cage aggression in group-housed male mice is a major welfare concern and may compromise animal research. Conventional cages prevent flight or retreat from sight, increasing the risk that agonistic encounters will result in injury. Moreover, depending on social rank, mice vary in their phenotype, and these effects seem highly variable and dependent on the social context. Interventions that reduce aggression, therefore, may reduce not only injuries and stress, but also variability between cage mates. Here we housed male mice (Balb/c and SWISS, group sizes of three and five) with or without partial cage dividers for two months. Mice were inspected for wounding weekly and home cages were recorded during housing and after 6h isolation housing, to assess aggression and assign individual social ranks. Fecal boli and fur were collected to quantify steroid levels. We found no evidence that the provision of cage dividers improves the welfare of group housed male mice; The prevalence of injuries and steroid levels was similar between the two housing conditions and aggression was reduced only in Balb/c strain. However, mice housed with cage dividers developed less despotic hierarchies and had more stable social ranks. We also found a relationship between hormone levels and social rank depending on housing type. Therefore, addition of cage dividers may play a role in stabilizing social ranks and modulating the activation of hypothalamic–pituitary–adrenal (HPA) and hypothalamic-pituitary-gonadal (HPG) axes, thus reducing phenotypic variability between mice of different ranks.

## Introduction

Laboratory mice are typically group housed to account for their social needs [1], a requirement that is also mandated by law in many countries. To cope with enforced proximity with conspecifics and to avoid escalated aggression [2], both male and female mice form dominance hierarchies [3, 4]. In males, these relationships are formed through repeated agonistic interactions [5, 6], which are typically highest during dominance formation [7] and gradually decline as social ranks stabilize over time [8]. However, even in stable hierarchies, dominant animals continue to enforce their status through overt (chasing, biting and attacks) and covert (mounting, displacement) aggression [9]. The confines of the laboratory cage may prevent subordinate animals to escape from aggressors, leading to escalated aggression, stress, pain and injury. Aggression in male mice is one of the most common welfare concerns in laboratory mouse husbandry [10, 11], and wounding due to aggression is the second most common clinical condition in mice [12].

Several internal and external factors can affect the level of aggression and shape the type and stability of dominance hierarchy. Routine husbandry practices (such as cage cleaning or visual checks) can increase fighting [8, 13, 14] and disturb the dominance hierarchy [15], while the physical environment (such as cage structure and enrichment) has been shown to influence the type of dominance hierarchy formed [4, 16]. Mice in standard laboratory cages most often establish a despotic hierarchy [4, 17–19], where one individual has a defined alpha rank and whose cage mates have less defined subordinate ranks. Linear hierarchies, where individuals have unique social ranks (e.g. alpha, beta, gamma), are more often seen in complex vivaria with nest boxes, that more closely approximate the natural habitat of mice [20–22]. Different methods have been adopted at research facilities to minimize aggression, such as: housing littermates, transferring nesting material during cage changes, group size of five mice per cage, lower room temperature and absence of environmental enrichment [11, 13, 14, 23–26]. However, despite these recommendations, aggression often persists [11].

Aggression, pain and social stress can also affect the phenotype [27] and cage mates of different social ranks can differ in behaviour and physiology [28–32]. Dominant animals initiate more fights and defend resources and territories, while subordinates inhibit escalated aggression, and these behaviours are supported by changes in physiology, including the hypothalamic–pituitary–adrenal (HPA) and hypothalamic–pituitary–gonadal (HPG) axes [33]. Typically, the dominant rank is associated with higher levels of circulating testosterone and lower levels of corticosterone, while subordinate animals exhibit physiological changes adapted to socially stressful contexts [31]. However, according to a recent meta-analysis [34] there is little evidence for systematic phenotypic differences between mice of different ranks and the direction of the relationship may depend on contextual factors, such as hierarchy type, stability, despotism and group size [33, 35, 36]. While elevated steroids in either dominant or subordinate mice may be beneficial in the short-term, they can lead to long-term health problems [29]. Thus, escalating and ongoing aggression and social defeat, highly despotic or unstable social groups all raise concerns about the welfare of group-housed mice, even in the absence of injury. In addition, working with animals that are severely socially stressed, may create additional variability that can reduce external validity and replicability of experimental results. Therefore, environments that reduce aggression might also have a direct (as a result of agonistic interactions) or indirect (via dominance type or stability) effect on the phenotype of mice of different ranks. Finding ways to decrease aggression and allow mice to form stable social groups will therefore not only minimize pain and social stress, but will also reduce the number of animals used in research.

A recent study showed that partial cage dividers can reduce aggression in Balb/c male mice [37], by separating the home cage into several compartments which may allow mice to avoid the aggressor. Here, we expanded on this work, by testing if long-term housing with partial cage dividers impacts not only aggression, but also wounding, in two commonly used mouse strains (one inbred, one outbred). Furthermore, we aimed to determine the role of cage dividers in modulating the relationship between steroids and social rank, by affecting the social structure (despotism) and the stability of social rank. We examined the relationship between fecal and hair steroid levels with social status in mice housed either with or without cage dividers.

## Material and methods

This study was carried out in accordance with guidelines of the Swiss Animal Welfare Ordinance (TschV 455.1). It was approved by the Cantonal Veterinary Office in Bern, Switzerland (permit number BE 5/21). The reporting of the study follows the ARRIVE 2.0 guidelines for reporting animal research [38].

### Animals and housing

**Fig 1:**
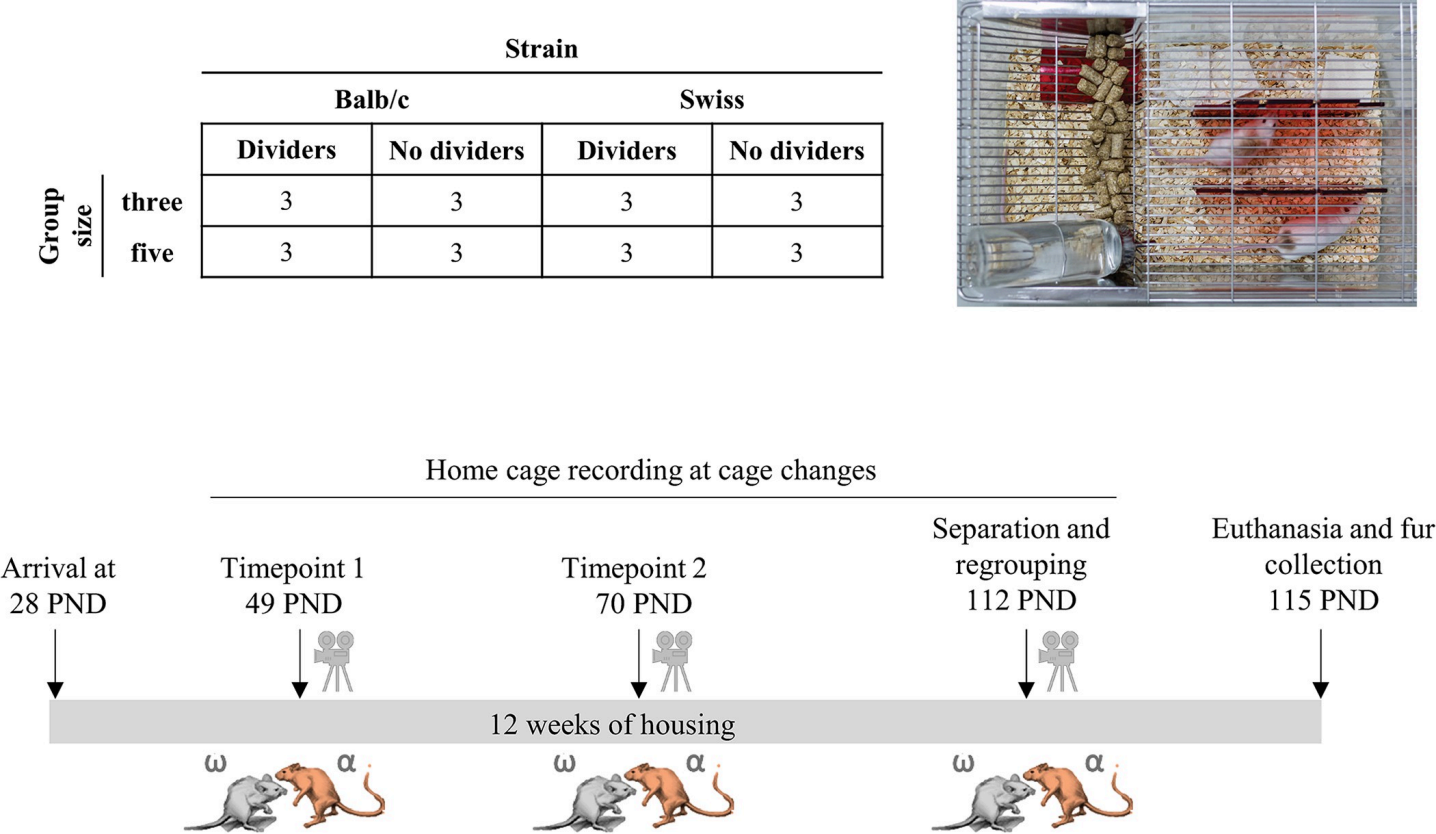
Timeline and experimental design. A total of 96 male Balb/c and SWISS mice were housed in groups of three and five either in cages with or without cage dividers. Cages were changed once weekly, and at week three (PND 49) and six (PND 70) of housing, home cages were recorded one day before and after cage change, to measure aggression. At week 12 of housing (PND 112), animals were singly housed for 4 hours and then placed in fresh cages, after which cages were recorded to measure aggression. After 12 weeks of housing, animals were killed and fur was collected from dorsal part to measure corticosterone and testosterone metabolites. Mouse figures were made in Dall-ee2. Photograph by Adrian Herrera.

48 male RjOrl:SWISS (SWISS) and 48 Balb/cJ (Balb/c) mice aged four weeks were purchased from Janvier Laboratories, France. All mice were weighed at arrival and housed in groups of three or five, balanced for body weight. All mice were assigned individual IDs and marked on their fur with a black non-toxic marker (Stoelting Co.). Cages were randomly assigned to housing with or without cage dividers using the randomization tool on (random.org). All mice were housed in type III cages (Tecniplast) with woodchip bedding (Lignocel Safe), two nestlets (Plexx) and a transparent red polycarbonate tunnel (Datesand). Half of the cages also included two red transparent cage dividers made from acryl (4 mm thick). The dividers divided half of the cage into three compartments, while the food area remained open. There was also a gap on the side opposite the food hopper, where the animals could move between the three compartments, so there were no dead ends (Fig 1). Cages were allocated to the rack so that housing, strain and group size were balanced across the rows and columns.

Animals were housed under 12:12 h reversed dark:light cycle, with room temperature ranging between (21–23 ^ο^C) and relative humidity (30–65%), with *ad libitum* food (Kliba Nafag, #3430) and tap water.

### Experimental design and data analysis

We used a fully crossed 2 (housing) x 2 (strain) x 2 (group size) design. With the two housing groups, two strains and two group sizes, the set up resulted in eight different conditions and a sample size of n = 24 cages, which was the smallest possible sample size according to Mead’s resource equation [39]. Mice were housed for a 12 week period, during which cages were changed once per week. Statistical analyses were carried out using R 3.6.0 [40].

### Wounds and injuries

Mice were inspected for wounds daily at cage checks and individually once per week during cage changes. Each mouse was given a score ranging from 0–5, as defined by [41]. Briefly, if a mouse was given a score 1, it was monitored daily to see if wounding escalated. If a mouse was given a score 2 or more, it was removed from the home cage and euthanized. Outcome measures were number of cages affected and the total number of injured mice. The effect of housing on the number of cages that were affected by injured animals was analysed with a Fisher-exact Test.

### Behavioural observations

Cages were recorded (Vivotec infrared sensitive cameras) at three time points to assess aggression and space use: 1^st^ and 2^nd^ time points were at weeks three and six of housing, before and immediately after cage changes, and the 3^rd^ time point was after a four hour separation period at the beginning of the dark (active) phase, during which cage mates were singly housed in type III cages with woodchip bedding (Lignocel Safe), two nestlets (Plexx) and *ad libitum* food (Kliba Nafag, #3430) and tap water. After four hours all cage mates were housed together in a new home cage and cages were recorded. At all three time points, cages were recorded for 24h before cage change, after cage change and after isolation housing. All videos were coded using Mangold Interact by two coders (CS and AH). To assess the frequency of agonistic interactions, 30 min of each of the first six hours of the dark phase was coded, one day before cage change and immediately after cage change (six hours of observations per cage per time point, 15 hours of video observations per cage in total). Using all occurrence sampling, the observers coded all chases, bites, mounts and attacks as well as the ID of the aggressor and target mouse and location of interaction. Individuals that directed biting, fighting, chasing or mounting were considered winners of each interactions. Individuals that exhibited fleeing or subordinate posture were considered losers of the interaction. If there was no clear winner (if the target mouse fought back), the event was recorded as a tie. Ten percent of the recordings were coded by both coders for inter-rater reliability and 10% of the recordings were re-coded by the same coder for intra-rater reliability. Inter-rater reliability for the different behaviours was moderate (Cohen’s Kappa ranging from 0.50 to 0.78), therefore the overall number of agonistic interactions were used for the analysis (Cohen’s Kappa = 0.85). Intra-rater reliability was 0.66 and 0.98 (Cohen’s Kappa) [42, 43].

For baseline aggression levels, all agonistic events during the two time points (1^st^ and 2^nd^) and before and after cage changes were pooled together for analysis. The numbers of agonistic events (dependent variable) were analysed using a general linear model *glm*.*nb* with a log link function of the MASS package [44]. The assumption of a negative binomial distribution of the data was examined and verified graphically to validate the chosen statistical approaches. Number of fights is reported as relative to number of mice in cage. Time point (1^st^ and 2^nd^), before and after cage change, strain, group size and housing (with or without dividers) were included as fixed factors. All interactions with the housing were also included in the model.

Effects of housing on the number of agonistic interactions after separation housing were analysed separately, as the context of isolation housing was different to routine cage changes and because events were only coded after the reunion of cage mates. Number of fights is reported relative to number of mice in cage. Again, the data were analysed using the *glm*.*nb* function, with strain and housing as fixed factors and their interaction included in the model. From the 2^nd^ time point until the end of experiment, many mice were excluded from the study due to wounding, resulting in group sizes from two to five mice per cage. Therefore, group size was excluded from the model.

Location of fights was analysed using the *glm*.*nb* function, with relative number of agonistic events at food hopper as dependent variable, and time point, before after cage change, strain, group size and housing, and interactions with housing as fixed factors. Here, locations of all agonistic interactions across all three time points were pooled together.

### Dominance hierarchy

The total frequency of wins and losses accrued by each individual was aggregated into separate frequency win/loss sociomatrices for each cage. Social dominance ranks were calculated for each cage using *Elo* package [45]. For each cage, the following measures characterizing the dominance hierarchy were calculated: despotism score, social dominance ranks of each mouse and rank stability. The level of despotism tells us how each alpha male monopolizes agonistic interactions within the cage, and was assessed by determining the absolute difference in win proportions between alpha and subordinate males for each cage. Social groups with despotism scores higher than 0.5 are considered to be highly despotic whereas groups with despotism scores lower than 0.5 are considered to have low despotism [33]. Effect of housing on despotism was analysed using the *glm* function, with housing, group size and strain as fixed factors. Dominance ranks of animals in each cage were determined by calculating a David’s Score (DS), which measures the individual proportion of wins. Briefly, each individual is given a weighting value based on the summed total of their agonistic success against each individual. The normalized David’s score is then calculated by subtracting the unweighted and a weighted sum of an individual’s proportion of losses to the sum of wins and losses from the unweighted and a weighted sum of their proportion of wins for each dyad [46]. The normalized David’s score corrects for the likelihood that the interaction occurred by chance. Rank stability between basal social ranks (calculated form agonistic interactions during the 1^st^ and 2^nd^ time points) and social ranks after disturbance (after isolation housing) were calculated with Spearman partial correlations of normalized David scores, controlling for cage. Effect of housing on rank change after isolation housing was analysed with a linear mixed effect model from the *lmer4* package [44], with DS as dependent variable, strain, group size, housing and despotism as fixed factors, and mouse nested in cage as random factor. The distribution of residuals was inspected using frequency histograms and normal Q-Q plots to assess deviation from normality. With 5 mice in a cage, the DS ranges from −4 to 4, with a score of −4 representing a mouse who lost all fights and a score of 4 given to a mouse that wins all fights.

### Hair and fecal corticosterone and testosterone (metabolites)

Steroid metabolite levels in feces of mice represent a measure of steroid release over a few hours [47, 48], while hair steroids are thought to reflect the cumulative stress burden better than single fecal samples [49]. Fecal boli were collected from each individual mouse during the four hour isolation housing, therefore fecal steroid levels were analysed for each animal. All fecal boli from the isolation cage were collected and stored in Eppendorf tubes at -20 degrees C until further processing and analysis. Two days after isolation housing and reunion, mice were killed with intraperitoneal pentobarbital injection followed by decapitation. Fur was collected from the dorsal part of all mice using an electric shaver (Wahl, Super trim) and stored at -20 degrees C until hormone analysis. Effects of housing on fecal and hair corticosterone and testosterone metabolites were assessed with a linear mixed effect model from the *lmer4* package [44]. For each model, we checked the distribution of residuals using frequency histograms and normal Q-Q plots to assess any major deviation from normality. Hair corticosterone metabolite levels were *sqrt* transformed. To assess effects of housing on steroid levels, housing, strain, and group size, were added to the model as fixed factors and mouse nested in cage as a random factor. Interactions with housing were added to the model. To estimate effects of social rank on steroid levels, despotism level and social rank (dominant or subordinate), plus social rank x despotism interaction were added to the model. For fur steroid levels, 100 mg hair per sample and 5 ml 100% methanol were used for hair extraction. A total of 2.5 ml of each extracted sample was dried and re-dissolved in 0.5 ml EIA buffer. For processing of fecal samples see Touma et al. [50]. Briefly, fecal corticosterone metabolites were extracted by incubating feces in 96% ethanol overnight at a ratio of 5 ml ethanol per gram feces. Metabolite levels were measured with a well-established 5α-pregnane-3β,11β,21-triol-20-one enzyme immunoassay (EIA) [51]. A corticosterone EIA was utilized to measure hair samples [52], and a testosterone EIA for feces and hair [53]. All steroid analyses were performed at the University of Veterinary Medicine in Vienna, Austria, using in-house EIAs. Details of the EIAs including cross-reactions can be found in the above given references.

## Results

### Exclusion criteria and attrition rate

Total number of animals at the beginning of study was 96, split across 24 cages. Because of early termination of experiments due to pre-specified humane end-points (wounding), the number of cages varied across the study (See Table 1). Two cages were removed from experiment after three weeks of housing. Furthermore, nine cages had at least one mouse removed; therefore in some cages group size was reduced. Five Balb/c cages had the group size reduced from three to two mice, two Balb/c cages with five mice had the group size reduced to three and four, and two Swiss cages had the group size reduced from three to two mice. All removals of individual mice from affected cages happened after the 2^nd^ home cage recording. Therefore, the group sizes (three and five mice per cage) for the two home cage recordings (1^st^ and 2^nd^ time point) were not affected. Group sizes at isolation housing, fecal boli collection and fur collection were reduced for several cages (group sizes ranged from two to five mice per cage).

**Table 1:** Number of cages included in the study.

	Home cage recording TP1				Home cage recording TP2				Isolation housing and fecal boli collection			
	Balb/c		Swiss		Balb/c		Swiss		Balb/c		Swiss	
	Dividers	No dividers	Dividers	No dividers	Dividers	No dividers	Dividers	No dividers	Dividers	No dividers	Dividers	No dividers
Three mice	3	3	3	3	3	3	3	3	2	3	3	3
Five mice	3	3	3	3	2	2	3	3	2	2	3	3

Number of cages per strain and housing varied across the three data collection points as some cages were removed from the experiment due to wounding.

### Injuries

**Fig 2:**
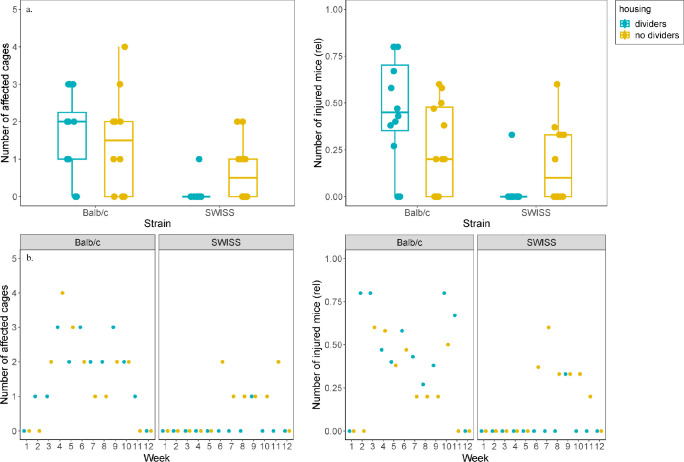
Number of affected cages and injured mice. Mice were checked for wounds daily at cage checks and once per week during cage change. a) Number of cages with injured mice and the overall number of injured mice were higher in Balb/c strain. Number of injured mice is reported as relative to group size (three or five). B) In Balb/c mice, wounding occurred across the 12 week housing period, while in SWISS mice most wounding was seen after six weeks of housing. Boxplots show medians, interquartile ranges and 95% confidence intervals.

Overall, nine out of twelve (75%) Balb/c cages were affected by wounding, five (83%) with dividers and four (67%) without dividers. 32 (67%) of all Balb/c mice were injured, 18 (56%) were from cages with dividers and 14 (44%) from cages without dividers. In the SWISS strain, five out of twelve cages (42%) were affected, one (17%) with dividers and four (67%) without. In total, only eight (17%) of all SWISS mice were wounded, one (12%) from a cage with dividers and seven (88%) from cages without dividers (Fig 2).

There was no difference in the number of affected cages between cages housed with or without dividers (Fisher exact test, χ^2^ = 0.51, p = 0.68). The effect of cage dividers on the number of injured individuals could not be tested statistically, as the sample size was not sufficient for a model including a random factor (the cage). Thus, only descriptive results can be provided for individual-level outcomes (Fig 2).

### Agonistic interactions

**Fig 3:**
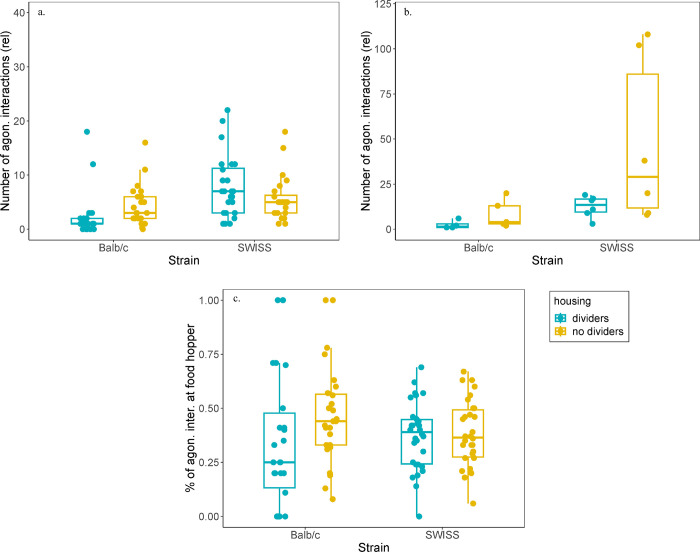
Relative number of agonistic interactions and space use. (a.) Level of agonism during the two cage changes (1^st^ and 2^nd^ time point) differed between strains and housing types. (b.) After isolation housing, mice housed without cage dividers tended to fight more. Number of fights was reported as relative to number of mice in cage. (c.) Across all observations (TP1, TP2 and after isolation housing), there was no effect of housing or strain on location where most agonistic interactions were seen. Boxplots show medians, interquartile ranges and 95% confidence intervals.

Two cages were excluded from the experiment due to wounding (Balb/c strain, one with and one without cage dividers), before the home cage recording. During the two time points, housing mice with cage dividers affected the level of agonism in both strains in opposite directions. Balb/c mice housed with dividers had fewer fights, while SWISS mice housed with dividers fought more often (-GLM, housing:strain interaction, β = -0.95, p = 0.01, adjusted for multiple testing, Fig 3A). Group size had no effect on the number of agonistic interactions (-GLM, β = - 0.34, p = 0.34, adjusted for multiple testing). SWISS mice also fought more than Balb/c mice (-GLM, β = 1.6, p = 0.01, adjusted for multiple testing).

After isolation housing, we found that mice housed without cage dividers fought even slightly more, though this increase was not significant (-GLM, β = 1.21, p = 0.08, adjusted for multiple testing, Fig 3B). SWISS mice also fought more than Balb/c mice (-GLM,β = 1.6, p < 0.01, adjusted for multiple testing).

There was no effect of housing (-GLM, β = 0.30, p = 0.11) or strain (-GLM, β = 0.07, p = 0.71) on location of fights across the three time points (Fig 3C).

### Steroids in feces and hair

**Fig 4:**
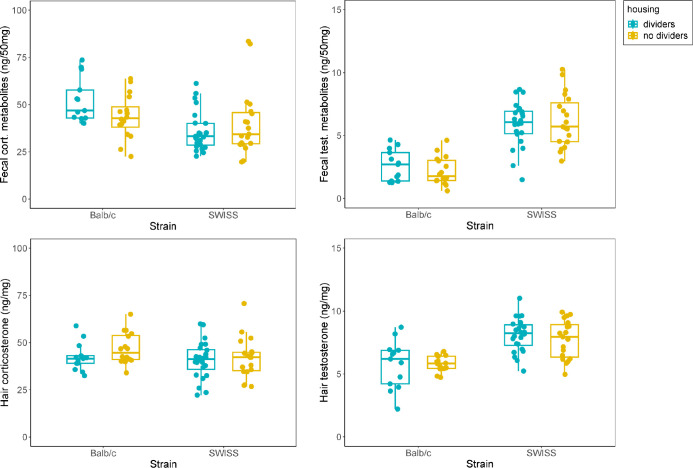
Steroids in feces and hair. Balb/c mice had higher fecal corticosterone metabolite levels, but the level of hair corticosterone was similar in both strains. Balb/c mice also had lower fecal and hair testosterone levels. There was no effect of housing on steroid levels. Boxplots show medians, interquartile ranges and 95% confidence intervals.

Housing with or without cage dividers had no effect on fecal corticosterone metabolite levels (GLMM, F_1,17_ = 0.21, p = 0.65), fecal testosterone metabolite levels (GLMM, F_1,17_ = 0.37, p = 0.55), hair corticosterone levels (GLMM, F_1,14_ = 1.88, p = 0.19) or hair testosterone levels (GLMM, F_1,15_ = 0.03, p = 0.86). Balb/c mice had higher fecal corticosterone metabolite levels (GLMM, F_1,17_ = 5.59, p = 0.03), lower fecal testosterone levels (GLMM, F_1,14_ = 36.52, p < 0.001) and lower hair testosterone levels (GLMM, F_1,15_ = 21.45, p < 0.001) than SWISS mice, while there was no strain difference in hair corticosterone levels (GLMM, F_1,13_ = 0.99, p = 0.34; Fig 4).

Fecal and hair corticosterone metabolite concentrations were not correlated (partial correlation, controlling for cage, r = 0.19, p = 0.11). Neither were fecal and hair testosterone levels (partial correlation, controlling for cage, r = 0.17, p = 0.16).

### Dominance hierarchy

**Fig 5:**
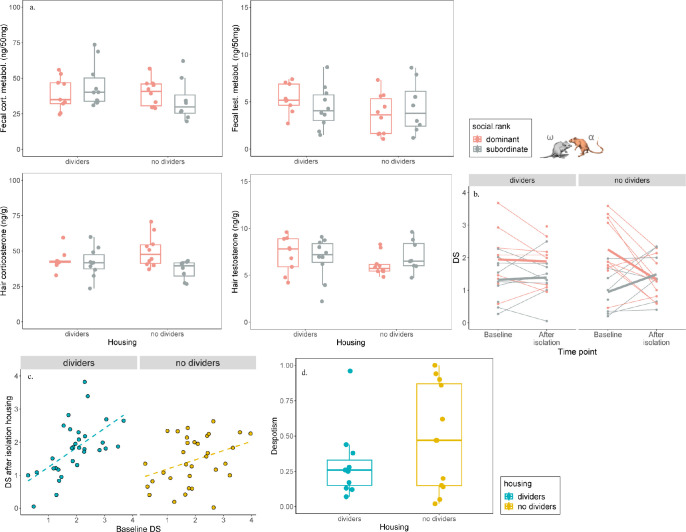
Level of despotism, stability of social ranks and steroid levels in animals of different ranks. (a.) In cages without dividers, dominant and subordinate males differed in hair steroid levels, but not fecal steroid metabolite levels. (b.) In cages with dividers, normalized David scores (DS) of dominant and subordinate males were the same during housing and after isolation housing, while in cages without dividers, in both dominant and subordinate males DS changed after isolation housing. (c.) DS of all mice in a cage calculated from 1^st^ and 2^nd^ time points were positively correlated to DS calculated after isolation housing in cages with dividers, but not with cages without dividers. (d.) Dominant males were more despotic in cages without dividers. Boxplots show medians, interquartile ranges and 95% confidence intervals.

Cages with dividers were different from cages without dividers in several aspects of the animals’ dominance hierarchy. Power was more evenly distributed in mice housed with cage dividers, as alpha males in these cages were less despotic (won fewer conflicts relative to subordinate cage mates) than alpha males in cages without dividers (despotism score 0.30 ± 0.07 and 0.48 ± 0.10, respectively; GLM, β = 0.42, P = 0.03, Fig 5D). Dominance ranks remained stable in cages with dividers. That is, basal ranks (normalized David’s scores calculated from 1^st^ and 2^nd^ time point) were positively correlated to ranks after isolation housing in cages with dividers (partial correlation controlling for cage, r = 0.62, P < 0.001), but not in cages without dividers (partial correlation controlling for cage, r = 0.27, P = 0.14, Fig 5C). David scores of both dominants and subordinates changed after isolation housing in cages without dividers, but not in cages with dividers (housing:time point interaction: GLM, β = 0.76, P = 0.02, Fig 5B).

### Dominance ranks and steroids

In cages without dividers, dominant and subordinate males differed in levels of hair steroids. Dominant mice had higher level of hair corticosterone (GLM, social rank:housing interaction, β = - 1.29, P = 0.004) and lower levels of hair testosterone (GLM, social rank:housing interaction, β = 1.47, P = 0.04) compared to subordinate mice, while there was no difference in cages with dividers. We found a trend of housing:social rank interaction in the opposite direction (GLM, β = 1.68, P = 0.07), but no main effect of housing (β = - 1.35, P = 0.10) or rank (GLM, β = - 0.58, P = 0.39) on fecal testosterone metabolite levels and no main effect of rank (GLM, β = 3.02, P = 0.59) or housing (GLM, β = - 1.46, P = 0.80) on fecal corticosterone metabolite levels (Fig 5A).

## Discussion

The main aim of this study was to assess if long term housing with partial cage dividers impacts aggression, wounding, social structure and stability of social rank in two commonly used mouse strains. We found that the addition of cage dividers reduced the frequency of agonistic interactions in Balb/c, but not in SWISS mice, even after an extended period of housing. These data partially support previous reports that cage dividers can mitigate aggression in laboratory mice, but these effects seem to be strain dependent [37]. Interestingly, while injuries started to appear only after the second week of housing for Balb/c mice, the addition of cage dividers did not reduce the prevalence of wounding across the two month housing period. Dominance hierarchies usually form when there is competition for resources such as access to mates, food, or territory [54, 55]. However, even in small groups of male mice living in standard laboratory housing, one animal will become the alpha dominant male within days, while the rank order of mid-ranking and lower-ranking individuals are resolved shortly thereafter [4, 20]. Therefore, across time, the aggression decreases [7, 56] as animals establish a social hierarchy within a group. This was also reported by Tallent et al. [37] who found that number of fights in Balb/c males was reduced already by the second day after housing, and this reduction was greater in mice housed with cage dividers. While the number of agonistic interactions after long-term housing in our study was relatively low, the effect of cage dividers reducing aggression was only seen in Balb/c mice. Behaviours used to establish and maintain hierarchies generally involve overt (chasing, biting, attack) and more ritualized behaviours (mounting) that serve as signals of social status. It is possible that in Balb/c mice, housing with cage dividers reduced some of these more ritualized behaviours and not behaviours that led to wounding, such as biting. Hence, the prevalence of wounding between the two housing conditions was similar. Because inter-rater reliability for each agonistic behaviour was poor, we could not analyse these behaviours separately. Difference in agonism between Balb/c and SWISS mice is not surprising as laboratory mouse strains and substrains differ in various aspects of behaviour, including aggression [11, 57]. Balb/c mice can also become increasingly aggressive with time [58], despite reports that aggression declines once hierarchies are established, which is also supported by our finding that injuries and fighting in this strain were seen across the two month period. Interestingly, we found no evidence that aggression increased with increasing group size as reported by other studies [26]. Altogether, our data confirm what has been pointed out in previous review articles on aggression in male mice: that the problem is complex and that it is unlikely that one solution will fit every situation [58].

Mice housed with cage dividers were not less stressed, as measured by fecal and hair steroid levels. Thus, even in cages with decreased agonism across the housing period, steroid levels on a group level were not lower. Testosterone, which is the main sexual steroid in male mice, is involved in the modulation of aggressive behaviour [59] while corticosterone is the primary stress hormone [60]. Corticosterone levels are affected by physiological or psychological stressors [61, 62] such as social conflicts, and can have direct implications for health [63]. Steroid metabolites are excreted via feces and urine, but they can also be measured in hair [48, 64]. Both fecal steroid metabolite levels [64] and hormone levels in hair can be used as a retrospective biomarker for long-term HPA and HPG activity due to environmental or physiologic stressors [49], however analysis of fecal steroid metabolites from one sample reflects a more acute stress response, while levels in hairs appear to be an appropriate marker of ongoing physiological stress [65]. In general, although aggression is the behaviour of concern in laboratory mice and both corticosterone metabolites and injuries serve as proxies for stress, we cannot conclude that mice housed without cage dividers had impaired welfare, even if they were more aggressive.

Cage environment not only affects aggression but can also shape the type and stability of hierarchies that develop. One such example is the level of despotism, that is, the extent of power of the dominant male, which can vary in different environments. Standard laboratory cages primarily display highly despotic hierarchies [4, 6, 17, 19, 20], where the dominant alpha male can win 80% or more of all fights, while the ranks of other mice are less clear [66]. In groups housed in more complex vivaria there is much more extended competition for dominant ranks [4] and these groups often develop more linear hierarchies, with each animal having a unique social rank [33]. Social ranks can also change, and even males that had been dominant for several weeks may lose this ranking abruptly [6, 67]. Despotism was relatively low in both housing groups, however we found that mice housed with cage dividers were less despotic and their ranks remained stable after cage groups were disturbed during isolation housing. Thus, housing mice with partial cage dividers may provide a possible solution to mitigate escalated aggression that can emerge from the conventional open space laboratory cages, which may prevent establishing a functional dominance hierarchy. We aimed to further elucidate if the compartmentalized cage design influenced where agonistic interactions took place. In complex vivaria with multiple floor levels and nest boxes, dominant alpha males typically patrol the top part of the vivarium, forming a territory surrounding the location of food, which is also the area where most fights occur [4]. In standard laboratory cages, dominant male mice also rest away from cage mates [19, 20], and mice who are frequently targeted by an aggressor have been shown to actively avoid them, particularly in despotic hierarchies [21]. We would therefore expect that addition of cage dividers would affect the location of fights, as subordinate animals would have more opportunities to avoid dominant males. However, we found no evidence for this. About half of the fights happened at the food hopper area and half in the compartments. We also tried to quantify spatial distribution of animals within the cage in more detail, however, as mice often transferred nesting material into one of the compartments, identification of animals was not always possible.

Corticosterone and testosterone metabolite levels can be correlated with social rank and phenotypic differences between dominant and subordinate animals become exacerbated following dominance hierarchy formation [28, 68]. Across species, elevated plasma testosterone is positively associated with dominant behaviours that enable individuals to attain and maintain high social status [33, 69, 70], while subordinate males have higher corticosterone metabolite levels [35, 63, 71, 72] and presumably experience a higher level of stress [73]. However, in laboratory mice there is no clear consensus regarding the relationship between social rank and steroid levels [34], and social context seem to be an important modulator of neuroendocrine output [16, 33, 74]. This is especially evident during times of social instability and increased fighting, which can lead to physiological changes of the HPG and HPA axis. During social ascent and challenge, there can be more testosterone surges in all animals [75], including subordinates [33], and dominant animals have higher corticosterone metabolite levels [76]. We found that dominant mice housed without cage dividers had higher hair corticosterone and lower testosterone metabolite levels compared to subordinate mice, which supports reports of social instability [3, 77]. The higher levels of testosterone found in subordinate males in cages without dividers may suggest that there is no suppression of testosterone production in these subordinate males by the dominant mouse. Dominant male mice also exhibit higher levels of locomotor activity [78] and patrolling behaviour [4] than subordinate males, so it is possible that the observed elevated corticosterone in dominant males housed without cage dividers is related to arousal of non-agonistic behaviour rather than a stress response related to social status conflict. Dominance ranks in our study were based on agonistic interactions. However, group housed mice develop complex social interactions that also include affiliative behaviours, such as grooming, sniffing and huddling, which can also affect HPA activity [79]. Several limitations of the present study should be taken into account before making generalisations based on these findings. In several cages, mice had bite wounds, and in some cages injured mice were removed from the study. Pain can activate the HPA axis [80] and the presence of injured mice may have masked the effect of housing condition on steroid levels. Additionally, removal of cage-mates can affect the social structure and ranks of remaining mice [3], which could have influenced the stability of social ranks. However, the same number of cages from both housing conditions were affected between the second video recording and isolation housing. It is therefore unlikely that the reduced group size influenced the difference in rank stability between the two housing conditions. Although the difference in steroid levels in our study was small, this further confirms findings that there is limited evidence for a clear difference between dominant and subordinate male laboratory mice without further examining social context [34].

## Conclusions

In summary, there is no clear evidence that housing male laboratory mice with partial cage dividers reduces aggression and wounding. Effects of cage dividers on aggression levels seem to be strain specific, making generalized conclusions difficult. Thus, while the cage dividers presented here may not directly improve the welfare of mice, they may play a role in affecting the social context and subsequently the steroid levels in mice of different social ranks.
